# Device Centric Throughput and QoS Optimization for IoTsin a Smart Building Using CRN-Techniques

**DOI:** 10.3390/s16101647

**Published:** 2016-10-06

**Authors:** Saleem Aslam, Najam Ul Hasan, Adnan Shahid, Ju Wook Jang, Kyung-Geun Lee

**Affiliations:** 1 Department of Electrical Engineering, Bahria University, E-8 Naval Complex, Islamabad 44000, Pakistan; saleem.aslam@bui.edu.pk; 2 Department of Electrical and Computer Engineering, Dhofar University, Salalah 211, Oman; nulhasan@du.edu.om; 3 Department of Information and Communication Engineering, Sejong University, Seoul 143-747, Korea; adnanshahid@nrl.sejong.ac.kr; 4 Department of Electronics Engineering, Sogang University, Seoul 04107, Korea; jjang@sogang.ac.kr

**Keywords:** Internet of Things, cognitive radio networks, channel scheduling, quality of service, smart building

## Abstract

The Internet of Things (IoT) has gained an incredible importance in the communication and networking industry due to its innovative solutions and advantages in diverse domains. The IoT’ network is a network of smart physical objects: devices, vehicles, buildings, etc. The IoT has a number of applications ranging from smart home, smart surveillance to smart healthcare systems. Since IoT consists of various heterogeneous devices that exhibit different traffic patterns and expect different quality of service (QoS) in terms of data rate, bit error rate and the stability index of the channel, therefore, in this paper, we formulated an optimization problem to assign channels to heterogeneous IoT devices within a smart building for the provisioning of their desired QoS. To solve this problem, a novel particle swarm optimization-based algorithm is proposed. Then, exhaustive simulations are carried out to evaluate the performance of the proposed algorithm. Simulation results demonstrate the supremacy of our proposed algorithm over the existing ones in terms of throughput, bit error rate and the stability index of the channel.

## 1. Introduction

With the recent advancements in sensor and communication technologies, the Internet of Things (IoT)-based network has become one of the most promising technologies for future applications. IoT offers a diverse range of applications from healthcare to surveillance [1,2,3,4]. IoT is a network of smart physical objects—devices, vehicles, buildings and other items—armed with different kinds of micro-controllers, transceivers and protocols for the dissemination of sensing and control information [5]. Therefore, IoT aims at provisioning an immersive, pervasive and ubiquitous connectivity of these smart objects. The current state of the art technologies to connect these heterogeneous objects include RFID, UWB, Bluetooth, ZigBee, WiFi Direct, etc. However, these technologies operate using the unlicensed spectrum band [6]. Therefore, the QoS over different established links is uncontrollable. However, the QoS is of prime importance for the IoT; hence, an innovative solution is needed to ensure the desired QoS. For this purpose, the cognitive radio (CR) technology is a promising solution in this scenario because it can exploit both the unlicensed and the licensed spectra for transmission [7,8]. Therefore, in this paper, our major focus is to optimize the throughput and ensure desired QoS to IoT devices using CR technology in a smart building environment.

The smart city is an emerging concept that brings benefits in the management and optimization of public services, such as transport and parking, lighting on roads and inside buildings, the surveillance and maintenance of public areas, the salubrity of hospitals and schools, etc. To achieve this, extensive and prevalent data availability and computing compatibilities are needed for different IoT devices [9,10]. The concept of the smart city starts with the idea of a smart building. A smart building consists of several control systems, such as ventilation, heating, air conditioning, communication, and many more [11,12,13,14,15,16,17]. To make intelligent decisions and effective optimizations of the available building resources, the data collected from IoT devices of different systems are all needed together. For instance, to make intelligent energy management decisions, the data collected from IoT devices belonging to different systems, such as heating, air conditioning and ventilation, need to be analyzed together. Therefore, a communication mechanism is required to provide the ubiquitous and pervasive availability of data collected by IoT devices of one system to the IoT devices of other systems within the building [2,4,18]. Other important concerns for IoT in a smart building are energy efficiency [19,20], admission control [21], naming and addressing [22], privacy and security [23], mobile sensing [24,25,26], etc.

The smart building is the convergence of construction, electrical and information technology [11,15]. It is a new concept of building construction with the aggregation of network systems and devices. These devices belong to different systems. Therefore, they have different QoS requirements in terms of data rate, latency and bit error rate. Additionally, these IoT systems consist of massive numbers of IoT devices [19,22]. Therefore, the integration of these devices must be done with higher spectral efficiency. One of the key technologies to improve spectral efficiency is cognitive radio (CR) technology [27,28]. Thus, we employ CR technology for these IoT devices. CR is an intelligent communication device that can learn, decide and reconfigure itself based on the available portion of the spectrum [29]. In a CR-based network (CRN), there are two kinds of users: (1) primary users (PUs) and (2) CR users or secondary users (SUs). CRs are only allowed to use the spectrum unused by the PU [30,31,32]. Without loss of generality, we keep the same terms of PUs and CRs for our cognitive IoT-enabled smart building system, i.e., the mobile users, IoT devices and networking monitors (NMs) deployed inside the building are referred to as CRs or SUs and the few mobile terminals as PUs.

The work in the domain of channel assignment of CRNs can be classified based on their architecture (centralized or distributed), allocation behavior (cooperative or non-cooperative), access technology (underlay or overlay) and scope (intra or inter) [33,34,35]. The research articles [27,34,35] are focused on the QoS provisioning in CRNs, but they are inclined towards cellular communication. In [36], an internetwork channel assignment scheme is presented to improve overall throughput at reduced interference. However, it ignores the QoS requirement desired by the PUs. Although the scheme presented in [37] considers the QoS parameters, it completely ignores the availability of the channel due to PU activity. In [38], the authors present a channel assignment scheme considering both the QoS parameters and PU activity. However, this scheme does not incorporate the traffic pattern for the applications running on different IoT devices. Similarly, the schemes presented in [39,40,41,42] target the energy efficiency, but they all lack in considering the PU activity and QoS of SUs. Likewise, the authors in [43,44] concentrated on congestion avoidance along with energy balancing, but do not consider individual QoS for optimal operation. Although the aforementioned schemes [33,34,35,36,37,38,39,40,41,42,43,44] are optimizing their objective functions, they are not suitable and applicable for the data dissemination of the IoT devices in a smart building environment. Hence, we propose a novel channel assignment scheme that considers the QoS requirements of different IoT devices, the availability of a channel due to the PU activity and the traffic patterns of different applications running on different IoT devices. The main contributions of our scheme can be summarized as follows:

- An optimization problem is formulated for the channel assignment to different SUs (i.e., IoT devices, mobile user) in a smart building environment. The objective is to serve the maximum SUs while satisfying their desired QoS.
- The optimization problem considers the number of QoS parameters (e.g., data rate, bit error rate and channel stability index) while taking into account the availability of channels by the PU activity and traffic patterns for different IoT applications.
- A novel particle swarm optimization-based algorithm is proposed to solve the optimization problem.
- To evaluate the performance of the proposed algorithm, exhaustive simulations are carried out by varying different parameters (e.g., the number of channels, the number of devices under scenarios where each IoT device uses different applications).

The rest of the paper is organized as follows. Section 2 describes the system model. Section 3 defines the problem formulation steps. Section 4 illustrates the details about the PSO implementation. Simulation results are presented in Section 5. Finally, Section 6 concludes the findings and sets the future directions.

## 2. System Model

**Figure 1:**
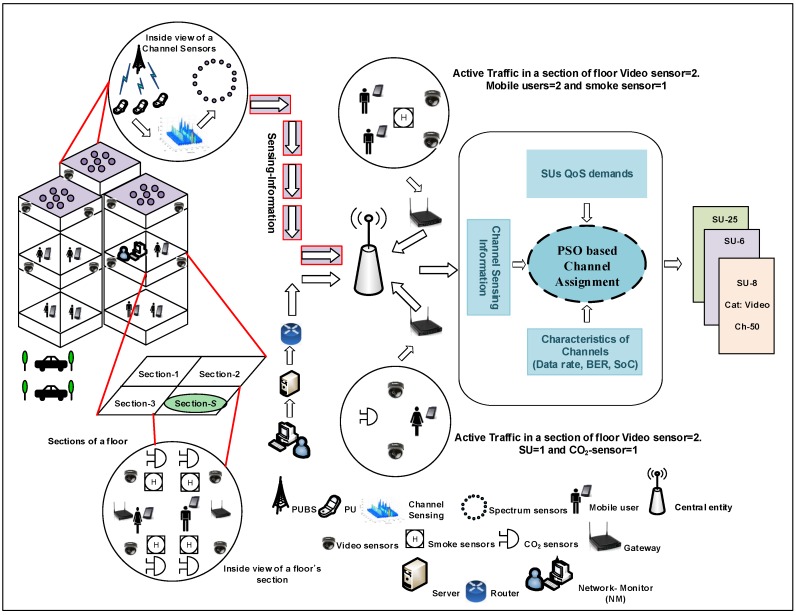
Proposed system model.

We consider a smart-building environment, which consists of a central entity, *Q* spectrum-sensors, *I* IoT devices, *M* mobile users and *N* free licensed channels. The central entity is responsible for spectrum-management, which includes the tasks of spectrum-sensing and spectrum-allocation. During spectrum allocation, the central entity allocates the available licensed channels to the IoT devices and mobile users. IoT devices and mobile users are considered to be SUs. However, they do not perform the task of spectrum-sensing because we employ dedicated sensors for the spectrum-sensing task. It not only helps to conserve the energy of IoT devices and mobile users, but also reduces the delay incurred in the spectrum-sensing process. First, the spectrum-sensors sense different channels to check whether they are empty or not and send their decisions to the central entity. Based on these decisions, the central entity decides finally whether a licensed channel is free or not. After spectrum sensing, the central entity performs the channel assignment to allocate the free channels to the IoT devices and mobile users. With reference to IoT devices, mobile users and networking monitors (NMs), we divided them into *C* classes. Each class has minimum requirements in terms of data rate ($\alpha_{min}$), bit error rate ($\beta_{min}$) and stability index ($\gamma_{min}$). A typical smart building with IoT devices, mobiles users and spectrum-sensors is shown in Figure 1.

**Figure 2:**
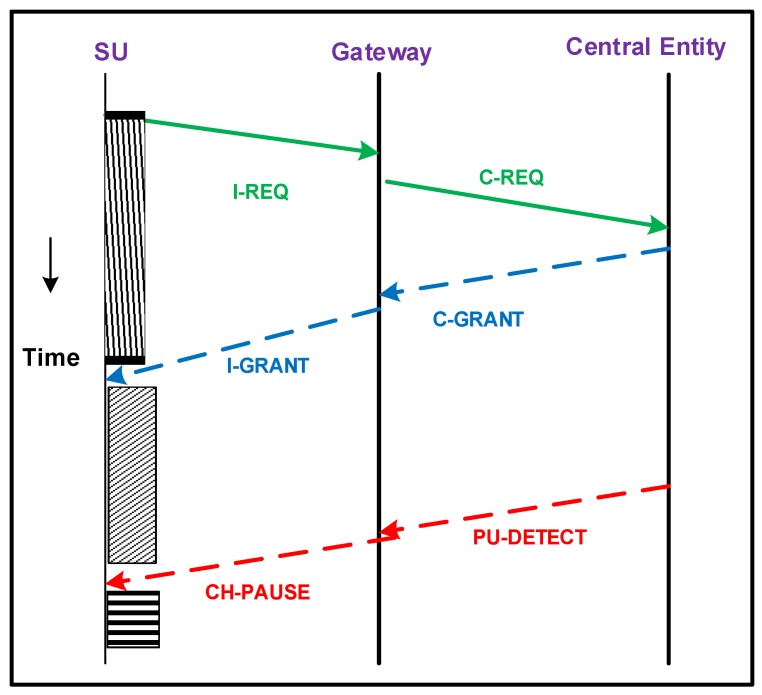
Frame exchange sequence between SUs, gateways and the central entity.

Since IoT involves energy-constrained devices, these cannot transmit over long distances; otherwise, their battery will drain out very quickly. Therefore, to facilitate their communication with the central entity, we divide the smart building into a number of sections. Each section has specific gateways. The communication between SUs and the central entity happens via gateways. The frame exchange sequence between the SUs, gateways and central entity is shown in Figure 2. A brief description of these frames is as follows. First, each SU that has data to send directs an I-REQmessage to its respective gateway. The I-REQ message specifies the class of a particular SU. After receiving I-REQs from all SUs, the gateways forward C-REQ message to the central entity. The C-REQ message includes the information about the requirements of all SUs associated with a particular gateway. After receiving C-REQ messages from all gateways, the central entity maximizes the total number of served users while assuring the minimal QoS requirement of each SU according to its class. For channel allocation to an SU, the central entity considers the data rate, bit error rate and stability index of the channel, which are discussed in subsequent subsections. The central entity broadcasts the C-GRANTmessage towards each gateway, which contains the list of channels assigned to each SU. Then, each gateway directs I-GRANT messages to the respective SUs. I-GRANT contains the channel number that is allocated to each SU. If a PU comes back on any channel, a PU-DETECTmessage is generated by the central entity to gateways, which indicates the arrival of the PU on the scheduled channel. As a result, gateways generates CH-PAUSEmessage to SUs to halt their ongoing transmission to avoid interference with PUs. Table 1 depicts the important symbols and notations for better understanding of the proposed scheme.

### 2.1. Capacity and Bit Error Rate

We assume that each SU belongs to a certain class c∈C and transmits the signal with power $\bar{P}$. Let $\rho_{k}$ be the interference experienced by an SU over the *k*th channel, and $\sigma_{k}^{2}$ is the noise content. Then, the achievable data rate $a_{k}$ of a *u*th SU over the *k*th channel can be computed using Equation (1) as follows [45]:

(1) $$a_{k}^{u}=\mu_{k}log\left(1+K\frac{{\bar{P}}_{k}^{u}}{\rho_{k}^{u}+\sigma_{k}^{2}}\right)$$

where $K=-1.5/log(5*b_{k}^{u})$ and $b_{k}^{u}$ is the BER of a *u*th SU over the *k*th channel. It is proven to be a good model for the modulation scheme, such as MQAMwith a constellation size greater than or equal to four [46].

### 2.2. Spectrum Sensing

Since SUs are using CR technology, the separate spectrum-sensors are deployed on top and inside the smart building, which are responsible for detecting the vacant spots in the spectrum for SUs. The transmitter detection (the signal received by the spectrum-sensors)-based hypothesis model can be given by Equation (2) as follows [28]:

(2) $$r_{k}\left(t\right)=\left\{\begin{array}{cc} \sigma_{k}^{2}\left(t\right) & ifH_{0}\\ \bar{s}\left(t\right)h\left(t\right)+\sigma_{k}^{2}\left(t\right) & ifH_{1} \end{array}\right.$$

where $r_{k}\left(t\right)$ is the signal received by a spectrum-sensor on the kth channel, $\bar{s}$(t) is the transmitted signal of the PU, *h*(t) is the channel response and $\sigma_{k}^{2}\left(t\right)$ represents the noise content. For the sake of simplicity, we employ a double threshold-based energy detector, which calculates the energy of a PU signal on the *k*th channel as follows:

(3) $$E_{k}=\sum_{y=1}^{Y}{\left|r_{k}\right|}^{2}$$

if the value of $E_{k}$ is greater than threshold-1, it is assumed that the PU is present over the *k*th channel, and the SU cannot use the *k*th channel for its transmission. However, if the value of $E_{k}$ is less than threshold-2, it declares that the channel is available to SUs.

### 2.3. Channel Stability Index

**Figure 3:**
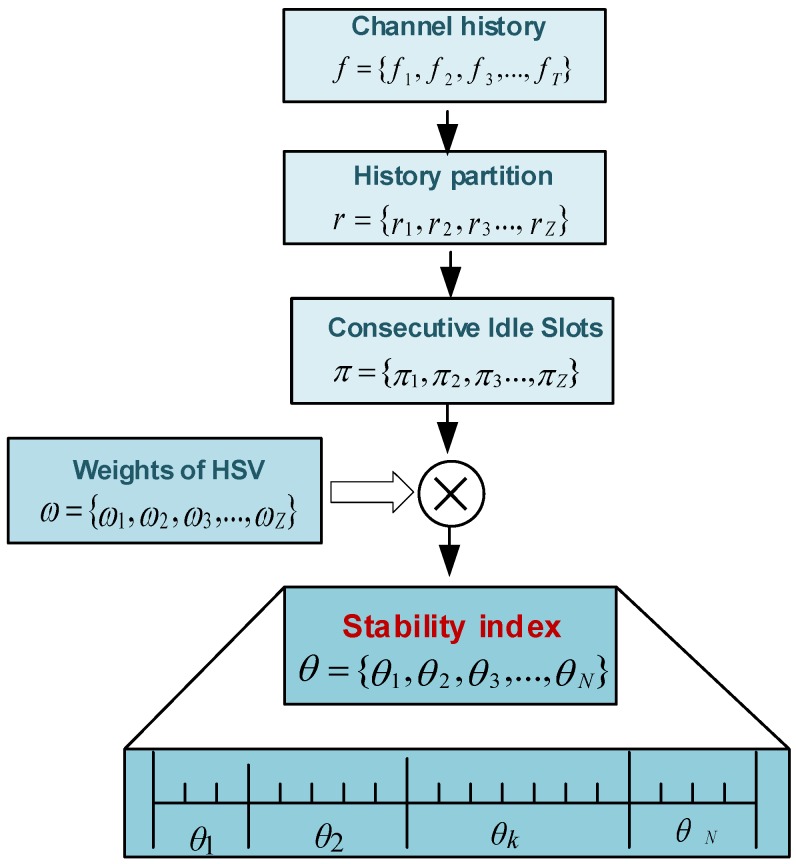
Stability-index calculation framework.

As mentioned earlier, separate spectrum-sensors are deployed for PU detection. Each spectrum-sensor creates a vector on each sensing interval and forwards it directly to the central entity, which characterizes the channels based on their stability (PU activity) index. A channel with the least PU activity is considered to be the most stable channel. Let $f_{k}$ = {$f_{1},f_{2},\ldots ,f_{T}$} be a history status vector (HSV) indicating the PU on-off activity over the *k*th channel during *T* time slots. For better prediction of PU activity, we vertically divide the HSV into different regions where z=1 represents the most recent and z=Z indicates the oldest region of the HSV, as shown in Figure 3. Suppose that $\pi_{k}$ = {$\pi_{1},\pi_{2},\ldots ,\pi_{Z}$} represents the consecutive number of free slots in different regions of HSV and that $\omega_{k}$ = {$\omega_{1},\omega_{2},\ldots ,\omega_{Z}$} indicates the weights assigned to each region of the HSV. The weights are assigned in decreasing order from the recent to the older samples of HSV. The stability index can be given using Equation (4) as follows:

(4) $$\theta_{k}=\sum_{z=0}^{Z}\omega_{z}\pi_{z}\;\forall z$$

where $\theta_{k}$ indicates the stability index for the *k*th channel. The larger value of $\theta_{k}$ indicates that the channel is more stable and that it has the least PU activity.

### 2.4. Traffic Classes of SUs

We classify the SUs (i.e., IoT devices, mobile users and NMs) into six different classes based on their QoS requirements, as shown in Table 2. The video IoT devices and video mobile users are considered in different classes due to different demands in terms of BER and the stability index of the channel (SoC). The smoke or fire detectors and ventilation control IoT devices need a small amount of data, but need more reliability and stability of the channel for real-time communication. The NM represents a real-time building monitoring class, which requires highly stable and reliable channels. For clarity, we use the letter *I*before IoT traffic class (e.g., Ivideo represents the video class for IoT devices). Similarly, the requirements of other classes are highlighted in Table 2 [47,48].

## 3. Problem Formulation

To provide the desired QoS to SUs within a smart building, it is desirable to find an optimal schedule for channel assignment in such a way that the maximum numbers of requests (members of different classes) are handled. Given the demands of SUs and available channels, an optimization problem is formulated that tries to maximize the number of served SUs at a particular time. Mathematically, it can be represented as follows:

(5) $$\begin{array}{cc} \mathrm{maximize} & F=\sum_{c=1}^{C}\sum_{u=1}^{U}\sum_{k=1}^{N}x_{k}^{u,c}\\ \mathrm{subject}\;\mathrm{to} & a_{k}^{u,c}x_{k}^{u,c}\geq \alpha_{min}^{c}\\ & \left(1-b_{k}^{u,c}\right)x_{k}^{u,c}\geq \beta_{min}^{c}\\ & \theta_{k}^{u,c}x_{k}^{u,c}\geq \gamma_{min}^{c,z}\\ & \sum_{l=0}^{\left|L\right|}x_{k}^{u,c}=0\;\forall \;l\neq k\\ & x_{k}^{c,u}=\left\{0,1\right\}\;\forall \;k \end{array}$$

where $x_{k}^{c,u}$ is a binary variable, which is either zero or one. If $x_{k}^{c,u}=1$, it means that the channel *k* is assigned to the *u*th user that belongs to class *c*. The optimization problem tries to maximize the summation of $x_{k}^{c,u}$ over the available channels, number of users and classes. Hence, it is equivalent to maximizing the number of served SUs. There are four constraints that ensure the minimum QoS requirements of an SU that belongs to a certain class. The first constraint makes sure that the selected channel meets the minimum data rate required by an SU. The second constraint guarantees the desired bit error rate. The smooth operation of SU over a channel is ensured via a third constraint by ranking and selecting the channels in terms of idle time. The last constraint ensures that a channel is used by an SU at a particular time instant. Algorithm 1 depicts the details about the PSO-based implementation of the proposed scheme. The computational complexity of Algorithm 1 is *O*(*Pξ*) [49], where *P* is the number of particles and *ξ* indicates the number of iterations to reach the global optimum.

**Algorithm 1** PSO-based device-centric QoS provisioning for SUs in a smart building.**Require:**
Available channels NTraffic classes *C*Minimum QoS of classes $\alpha_{min}^{c},\beta_{min}^{c},\gamma_{min}^{c}$SUs of a class *U*Acceleration coefficients $\eta_{1}$, $\eta_{2}$Random numbers $\delta_{1}$, $\delta_{2}$**Ensure:** Resource allocation ϕ[C×U,N]  **while** stoping criteria not meet **do**   **for** (*j*: 1 to *P*) **do**    **for** (*d*: 1 to *D*) **do**     Initialize position $S_{jd}$     Initialize velocity $V_{jd}$    **end**
**for**    Initialize particle personal best $Pb_{j}$    **if**
$Pb_{j}>Gb$
**then**     $Gb=Pb_{j}$ global best position    **end**
**if**   **end**
**for**   **for** (*j*: 1 to *P*) **do**    **for** (*d*: 1 to *D*) **do**     $V_{jd}^{\tau +1}=V_{jd}^{\tau }+\eta_{1}\delta_{1}\left(Pb^{\tau }-S_{jd}^{\tau }\right)+\eta_{2}\delta_{2}\left(Gb^{\tau }-S_{jd}^{\tau }\right)$     $S_{jd}^{\tau +1}=S_{jd}^{\tau }+V_{jd}^{\tau +1}$    **end**
**for**   **end**
**for**  **end**
**while**  **return**
*ϕ*

## 4. Particle Swarm Optimization

PSO is an artificial intelligence technique inspired from the social behavior of birds, and it is used to approximate the solutions for optimization problems involving a large search space [50]. An individual amongst the bird population is known as a particle, and it represents the possible solution (a bird in the flock) for a given problem. The fitness of each particle indicates how close it is from the ideal solution. The solution has definite boundaries, and the particles move within the *D*-dimensional boundaries (flying space for birds) [51]. Let *P* represent the total number of particles (birds or solutions) and $S_{j}^{\tau }=\left[S_{j1}^{\tau },S_{j2}^{\tau },\ldots ,S_{jD}^{\tau }\right]$ represent the position of the *j*th particle 1≤P≤D at iteration *τ*, where $S_{jD}^{\tau }$ indicates the position of particle in the *d*th 1≤d≤D dimensional space. At iteration *τ*, the velocity of the *j*th particle can be denoted as $V_{j}^{\tau }=\left[V_{j1}^{\tau },V_{j2}^{\tau },\ldots {,}_{jD}^{\tau }\right]$, which varies in the range $\left[-V_{max},V_{max}\right]$. During each iteration, the fitness of the particles is evaluated to indicate their merit. The position of particles depends on two key parameters: (1) personnel best ($Pb^{\tau }$); and (2) the neighbor best ($Nb^{\tau }$). Let $Pb^{\tau }$ indicate the best position of the *j*th particle and be the best position in comparison with the neighbors. If we consider the rest of the particles as neighbors, then this term can be called the global best, and it can be represented by $Gb^{\tau }$. These swarm parameters help with quick convergence towards the possible solution. The velocity and position of individual particles can be updated using the expression given in Equations (6) and (7) as follows:

(6) $$V_{jd}^{\tau +1}=V_{jd}^{\tau }+\eta_{1}\delta_{1}\left(Pb^{\tau }-S_{jd}^{\tau }\right)+\eta_{2}\delta_{2}\left(Gb^{\tau }-S_{jd}^{\tau }\right)$$

(7) $$S_{jd}^{\tau +1}=S_{jd}^{\tau }+V_{jd}^{\tau +1}$$

where $\eta_{1}$ and $\eta_{2}$ are two positive constants termed the learning factor or acceleration coefficients and $\delta_{1}$ and $\delta_{2}$ represent the uniform random numbers fully distributed in the range [0, 1]. The terms $S_{jd}^{\tau +1}$ and $V_{jd}^{\tau +1}$ represent the updated position and velocity factors for the *j*th particle, respectively.

### 4.1. Particle Encoding

The encoding process is one of the important steps in the PSO process. It assists in binding the particle with the solution. For the current scheme, the solution is an assignment of *N* channels to *M* mobile users, *I* IoT and network-monitors. For example, the encoding particles for the high-load traffic scenario is ($\Psi_{video}=5,\Psi_{voice}=5,\Psi_{web}=5,\Psi_{ivideo}=5,\Psi_{ico2}=5,\Psi_{NM}=5$) and for the low-load traffic is ($\Psi_{video}=2,\Psi_{voice}=4,\Psi_{web}=4,\Psi_{ivideo}=5,\Psi_{ico2}=4,\Psi_{NM}=1$). We perform the simulations using the aforementioned encoding scenarios, and we assume that mobile users, sensors and NMs are served on discrete channels.

**Figure 4:**
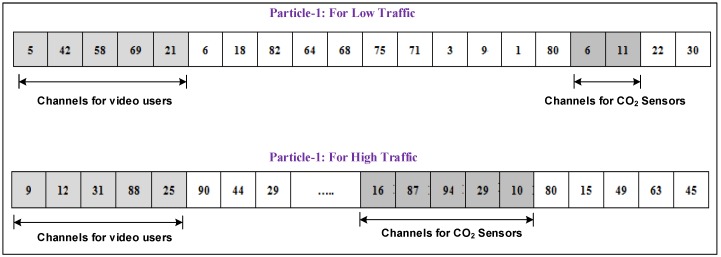
Particle encoding.

Figure 4 illustrates the encoding process for two traffic scenarios (i.e., low traffic and high traffic). The size of Particle 1 is 20, which indicates the total number of requests from mobile users, IoT and NM, i.e., $\Psi_{total}$ = 20, whereas the length of Particle 2 is $\Psi_{total}$ = 40. It is clear from the given scenarios that a particle represents the complete solution in terms of channel allocation to mobile users, IoT and NMs.

### 4.2. Position and Velocity Updates

To achieve the optimal solution, the swarm particles need to update their velocity and position. The velocity of the *j*th particle can be represented by the *D*-dimensional vector in which each element is a random real number, and it represents the change in channel allocation for a given iteration. For example, consider a particle for low load traffic; the velocity vector (−13 5 2 −8 33 24 −22 … −19 41 6 55) is added to the position vector (5 42 58 69 21 6 18, … , 6 11 22 30) to form the new position of the particle (8 47 80 61 54 30 4, … , 13 52 28 85). In order to bring the updated position of the particles within *D*-dimensional space, the velocities can be clamped using the given range $\left[-V_{max},V_{max}\right]$.

## 5. Performance Evaluation

In this section, we evaluate the performance of the proposed scheme using simulation results in MATLAB. In order to improve the clarity, the results are grouped into two subsections. Firstly, we present the simulation results for low traffic and high traffic scenarios to indicate the quick convergence of the proposed scheme towards an optimal solution. Secondly, the comparison is drawn between our proposed solution and existing schemes across (1) QoS parameters (e.g., data rate, BER and stability index) and (2) blocking probability. The impact of these evaluation parameters is investigated by varying the iterations, channels, traffic load and stability index (SoC). The Monte Carlo simulation model is considered to get average results over 500 iterations. PU traffic is modeled using on and off states with the PU arrival rate from 0.0 to 0.6. For the simplicity of SoC computations, the PU history is maintained for 30 slots, which are divided into three regions with weights $\omega_{1}=0.6$, $\omega_{2}=0.25$ and $\omega_{3}=0.15$, respectively. The rest of the simulation parameters are presented in Table 3 and Table 4.

### 5.1. Average Objective Function

**Figure 5:**
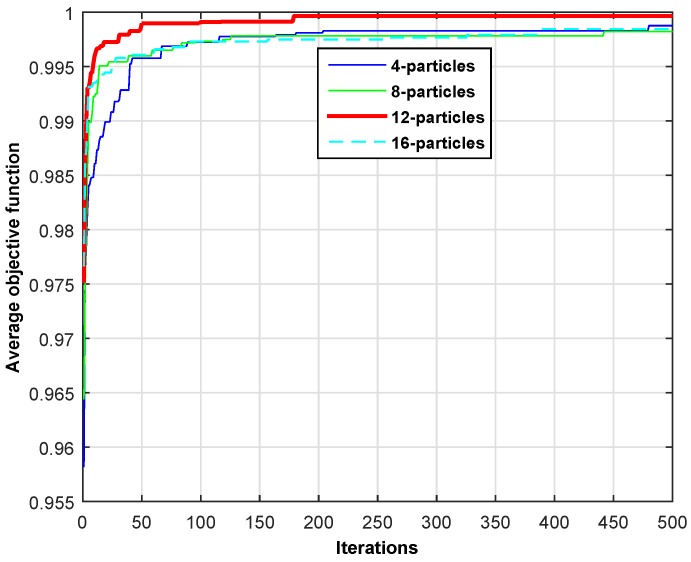
Average objective function for the low traffic scenario.

**Figure 6:**
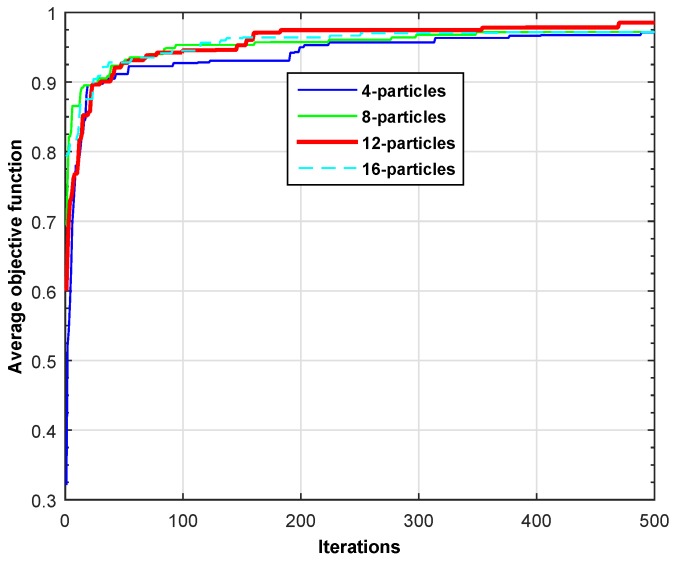
Average objective function for the high traffic scenario.

In this section, we present the simulation results to show the behavior of the objective function across different iterations. Figure 5 shows the objective function given in Equation (5) for four different particle sizes across different iterations. The PSO is an iterative algorithm that generally provides the best solution after a certain number of iterations. The stopping criteria (in terms of iterations) depend on the objective function and constraints of the given problem. We plot the results for low traffic and high traffic scenarios as depicted in Table 3. It is clearly visible through the two line plots that 12 particles is the most suitable population size for our problem. Therefore, the rest of the simulation results are carried out using a population size of 12-particles. In addition, the proposed scheme shows quick convergence towards the optimal solution for both scenarios. For example, the proposed scheme serves more than 95% of SUs’ requests for low traffic as shown in Figure 5 and approximately 90% of SUs’ requests for the high traffic case, as shown in Figure 6. Hence, the proposed scheme optimally manages the heterogeneous traffic of SUs in a smart-building.

### 5.2. Comparison with Existing Schemes

**Figure 7:**
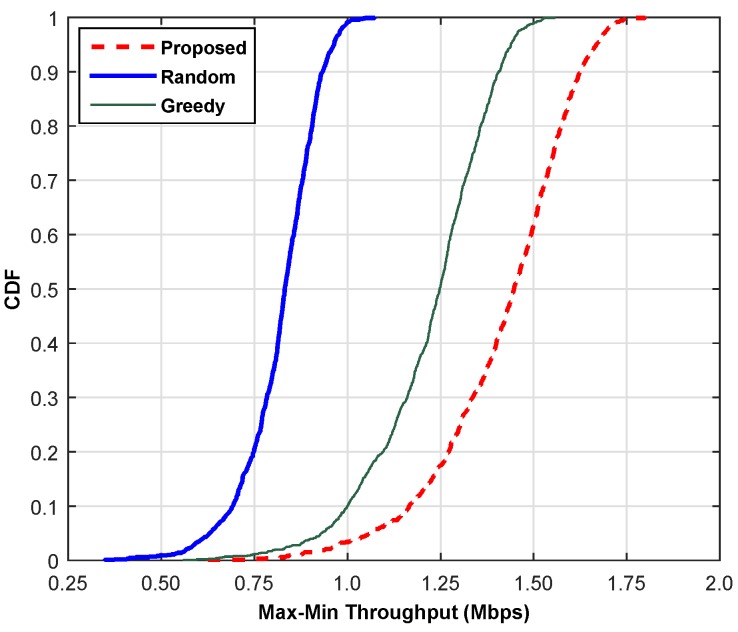
Cumulative distribution function of throughput.

**Figure 8:**
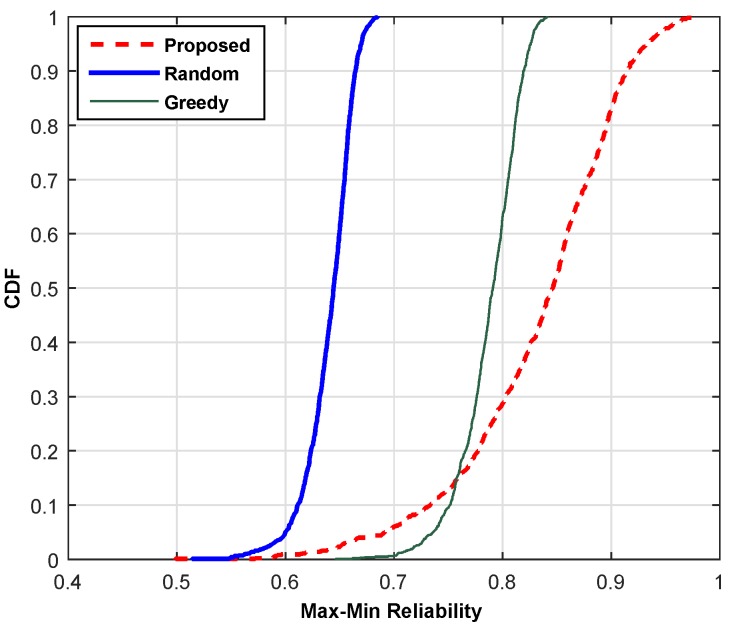
Cumulative distribution function of reliability.

This section compares the performance of the proposed scheme with the random channel selection (RCS) scheme and the greedy channel selection (GCS) scheme based on throughput, reliability and SoC. The RCS randomly chooses a channel from the available pool of channels with probability 1/*N* and allocates it to an SU without considering its QoS, whereas the GCS selects a locally-optimal channel for each user with the hope to reach a globally-optimal solution. The cumulative distribution function (cdf) plots are shown for (a) min-max throughput, (b) max-min BER and (c) min-max SoC. For the current comparisons, we select the high traffic mode and carry out the simulations at 500 iterations, and then, the average values are shown by adopting the Monte Carlo principle. Figure 7 compares the proposed scheme with the RCS and GCS schemes based on the overall throughput of the network. The combined throughput of SUs is plotted in comparison with the existing schemes to illustrate the performance gain of the proposed scheme. It is clear from the line plot that the proposed scheme provides approximately 75% and 16.25% higher throughput as compared to RCS and GCS, respectively. The proposed scheme can achieve throughput within the range of 0.74 to 1.75 Mbps, whereas RCS shows variation across 0.378 to 1.0 Mbps. Hence, the proposed scheme leads both schemes. The comparison based on reliability is illustrated in Figure 8. The result is simulated 500 times, and then, the average reliability index is plotted to show the performance gain of the proposed scheme. The RCS lags the proposed scheme in the reliability index by 39.68%. Although the GCS shows a leading pattern as compared to the RCS, it is still lagging behind the proposed scheme. Hence, our scheme is equally good for error-sensitive applications.

**Figure 9:**
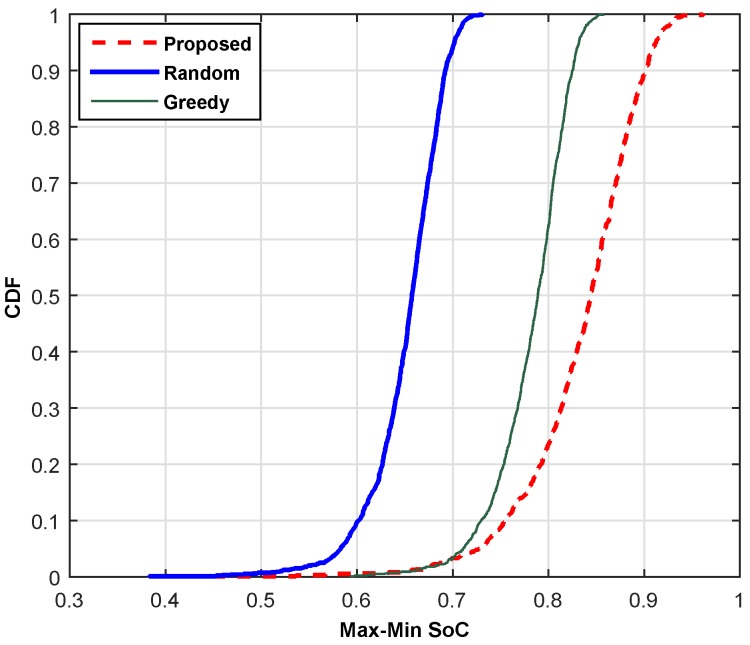
Cumulative distribution function of SoC.

Figure 9 highlights the performance gain of the proposed scheme across the min-max SoC. For the current simulation result, we plotted the average stability index acquired using different schemes at 500 iterations. The proposed scheme shows the leading behavior by selecting the channels with a higher stability index as compared to RCS and GCS. The higher SoC values are achieved through the mechanism depicted in Section 2.3. The proposed scheme selects the best channels in terms of least PU activity for the stable and smooth operation of SUs. Furthermore, it selects the channels within the range of 0.66 to 0.964 SoC, whereas the RCS shows values in the range of 0.52 to 0.72. Similarly, the range of SoC values acquired through GCS is higher than RCS, but lower than the proposed scheme. Hence, the proposed scheme provides the most stable channels to SUs.

### 5.3. Comparison Based on Blocking Probability

In this section, we compare the proposed scheme based on the combined blocking probability (i.e., 1-normalizedvalue of F) by varying the number of channels, SoC and traffic load. For these results, we consider the low traffic scenario and plot the blocking probability of SUs. Since there are 20 channel requests from SUs, at least 20 channels are required to provide wireless connectivity (there may be higher collisions if only 20 channels are available). However, the greater the availability of vacant channels, the lesser the blocking probability (collisions with the PU are reduced).

**Figure 10:**
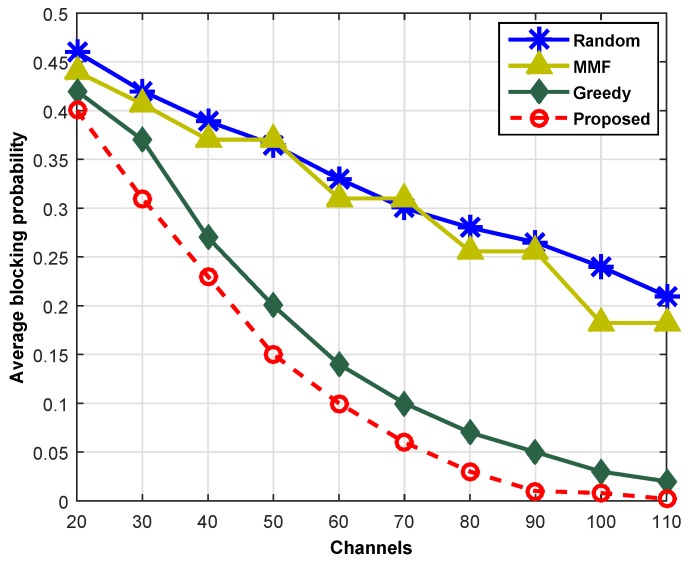
Blocking probability across different channels.

Figure 10 shows the blocking probability for different numbers of available channels and indicates that the blocking probability decreases with the increase of available channels. The proposed scheme shows significantly lower blocking rates as compared to the other schemes. For example, consider a case when *N* = 60 channels are available; the proposed scheme shows 40% less collision probabilities as compared to GCS, and it offers a three-times lesser blocking rate as compared to the RCS and max-min fair (MMF) schemes. The MMF scheme provides the same data rate to all SUs, regardless of their QoS requirements. With the availability of *N* = 100 channels, the proposed scheme shows a blocking probability of 0.01, whereas the blocking probabilities for GCS, MMF and RCS are 0.05, 0.25 and 0.265, respectively, which are significantly higher as compared to the proposed scheme. This comparison shows that if a large number of channels is available, then the proposed scheme accommodates almost all of the requests from mobile users, IoT and NM.

**Figure 11:**
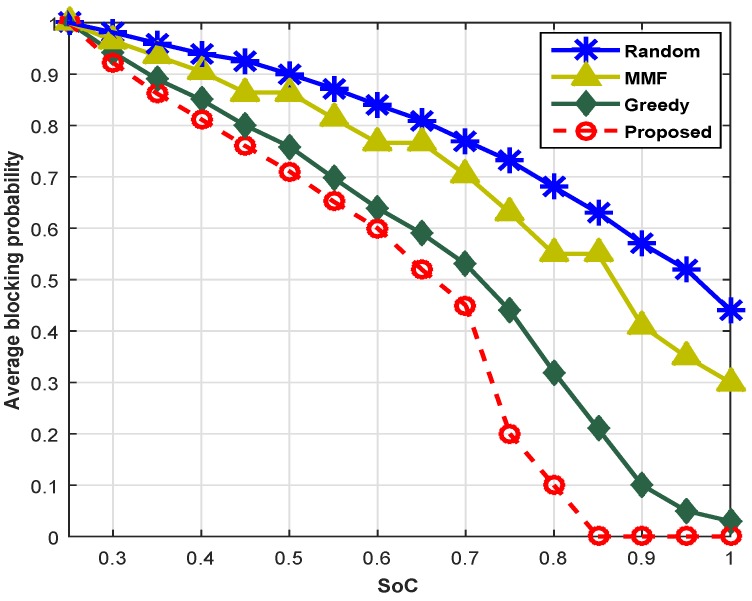
Blocking probability across different SoC.

Figure 11 compares the proposed scheme with the existing schemes based on the SoC. With SoC $\theta_{k}$ < 0.3, (i.e., the minimal SoC requirement of web traffic), not a single web-class SU gets the channel because the SoC cannot meet the minimum QoS requirement. However, when the SoC of available channels is greater than 0.3, the blocking probability decreases because the QoS demand of web users is satisfied, and they get the channel for different web services. Similarly, when the SoC becomes greater than 0.5 (i.e., $\theta_{k}$ > 0.5), the SUs that belong to video class get the chance for transmission, and the overall blocking probability reduces quickly. There is a sharp decline in the blocking probability of the network with the availability of channels with $\theta_{k}$ > 0.7. This is due to the fact that other classes receive service due to the availability of stable channels. Consider a case for $\theta_{k}$ = 0.7; the proposed scheme shows [17.7% 55% 71%] low blocking probability as compared to the GCS, MMF and RCS, respectively. Hence, the proposed scheme offers the better quality channels to SUs.

**Figure 12:**
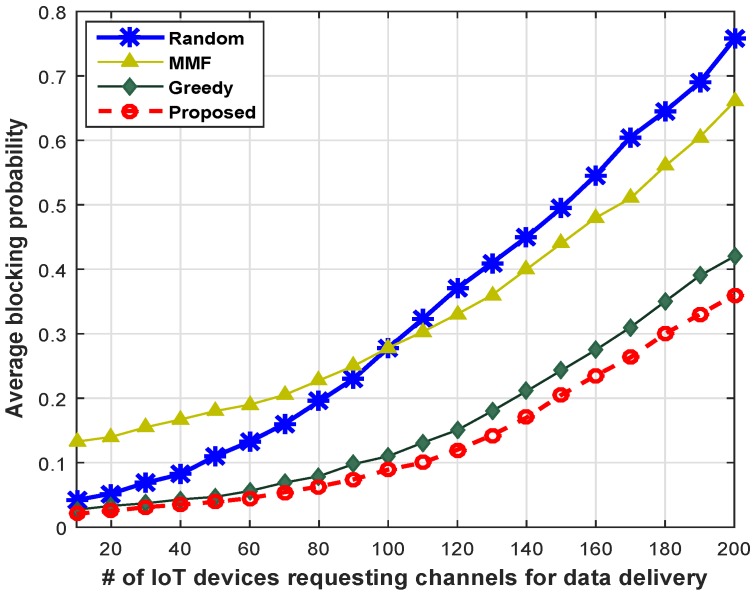
Blocking probability across different numbers of IoT devices.

Figure 12 compares the proposed scheme with the existing scheme in terms of IoT density. We vary the number of IoT devices belonging to different classes who are demanding channels for data transmission and plot the average blocking probabilities for different schemes. It is clear from the line chart of Figure 12 that the proposed scheme shows significantly lower blocking probability as compared to other schemes. For example, with 100 IoT devices that are requesting channels for data delivery, the proposed scheme shows 12.33% lower blocking probability compared to the GCS scheme and shows 31.66% less blocking probabilities in comparison with MMF and RCS, respectively. Hence, our scheme is well suited for the smart-building environment in order to cater to a large density of IoT devices.

The blocking probabilities of individual classes are presented in Table 5 for low and high traffic modes. The comparison is shown for four different classes, such as voice, web, Ivid (i.e., *I* indicates that it is a video IoT device) and NM. The web traffic of SU and the video traffic of IoT show higher blocking probabilities due to their stringent QoS requirements, as compared to the voice and NM traffic. Compared to GCS and RCS, the proposed scheme shows lower blocking probabilities for all traffic types. For example, consider the web traffic in low traffic mode; the proposed scheme demonstrates four-times and 1.5-times lower blocking probabilities as compared to RCS and GCS, respectively. Hence, the proposed scheme offers better support for heterogeneous traffic in a smart-building environment.

## 6. Conclusions

We develop a robust throughput and QoS optimization scheme for a smart building environment that allocates channels to mobile users, IoT devices and network monitors based on their required QoS. The results highlighted that the proposed scheme shows 39.68% and 12% to 33.8% better performance in terms of reliability (low bit error rate) and channel idle time, respectively. Considering the overall throughput of the network, the proposed scheme provides 16.25% and 75% higher throughput as compared to the greedy and random sharing schemes, respectively. Simulation results also demonstrate that the proposed scheme shows a significantly lower blocking rate for both low and high traffic modes compared to the existing schemes. Therefore, the proposed scheme can support a higher density of users with a competitive performance gain. In the future, we will extend the proposed framework for energy efficiency and incorporate the concept of RF energy-harvesting for IoT devices to achieve energy-balancing and support the green communication paradigm.

## Figures and Tables

**Table 1:** Symbols and notations.

Symbols	Meaning
F	Objective function
*N*	Available channels
*C*	Traffic classes
*Q*	Spectrum-sensors
*I*	IoT devices
*M*	Mobile users
*c*	Subscript of class
*u*	Subscript of the SU belongs to the *c*th class
*j*	Subscript of the swarm particle
*k*	Subscript of channels
*NM*	Network monitor
*HSV*	History status vector of a channel
*SU*	CR-based mobile user, IoT device or NM
*T*	Total history slots
$\bar{P}$	Transmission power of SU
*Z*	Partitions of HSV
*ω*	Weights for partitions of HSV
*E*	Energy of the PU signal used for spectrum sensing
*V*	Velocity of the swarm particle
*S*	Position of the swarm particles
*Pb*	Global best of the swarm particles
$\alpha_{min}$	Lower limit of data rate requirement
$\beta_{min}$	Upper limit of BER tolerance
γmin	Lower limit of the channel stability requirement
$a_{k}$	Data rate of the *k*th channel
$b_{k}$	BER of the *k*th channel
$\Psi_{c}$	Represents SUs of the *c*th class
$\theta_{k}$	Stability index of the *k*th channel
$x_{k}^{c,u}$	Stability index of the *k*th channel

**Table 2:** QoS parameters for different SUs. NM, networking monitor.

Classes	Minimal QoS Requirement		
	Data Rate (Kbps)	Bit Error Rate	Stability Index
	($\alpha_{\min}$)	($\beta_{\min}$)	($\gamma_{\min}$)
Video	90	5	0.5
Voice	9.6	10	0.75
Web	30.5	12	0.3
Ivideo	90	8	0.3
Ismoke, ICO${}_{2}$	5	10	0.8
NM	60	10	0.85

**Table 3:** Simulation parameters.

Parameters	Values
Power (P)	35 dBm
Noise variance ($\sigma^{2}$)	0.1∼0.65
Channels	100
PU activity	0.0∼0.6
Population size	12
PSO acceleration coefficients	2
PSO inertia weight	0.72
[−Vmax,Vmax]	[−100, 100]
Sensing interval	0.1 ms
Modulation scheme	MQAM
Constellation size	4
History slots	30
History partitions	3
Weights [$\omega_{1},\omega_{2},\omega_{3}$]	[0.6, 0.25 0.15]

**Table 4:** QoS parameters for different SUs.

Low Traffic		High Traffic	
Class	Members	Class	Members
Video	2	Video	5
Voice	4	Voice	5
Web	4	Web	10
Ivideo	5	IVideo	10
Ismoke, ICO${}_{2}$	4	ISmoke, ICO${}_{2}$	5
NM	1	ICo${}_{2}$	5

**Table 5:** Blocking probability of individual classes for low and high traffic.

Schemes	Traffic Classes of Mobile Users, IoT and NM							
	Voice		Web		Ivideo		NM	
	Low	High	Low	High	Low	High	Low	High
Random	0.2267	0.4760	0.2848	0.5510	0.3058	0.6251	0.0258	0.3989
Greedy	0.0834	0.1828	0.0935	0.2124	0.1060	0.2456	0.0098	0.1684
Proposed	0.0561	0.1124	0.0688	0.1396	0.0756	0.1567	0.0018	0.0995
